# Neuropeptide-inducible upregulation of proteasome activity precedes nuclear factor kappa B activation in androgen-independent prostate cancer cells

**DOI:** 10.1186/1475-2867-12-31

**Published:** 2012-06-20

**Authors:** Anna Patrikidou, Panagiotis J Vlachostergios, Ioannis A Voutsadakis, Eleana Hatzidaki, Rosalia-Maria Valeri, Chariklia Destouni, Effie Apostolou, Christos N Papandreou

**Affiliations:** 1 Department of Medicine, Institut Gustave Roussy, Villejuif, France; 2 Department of Medical Oncology, University Hospital of Larissa, Larissa, Greece; 3 Centre Pluridisciplinaire d’Oncologie, Centre Hospitalier Universitaire Vaudois, Lausanne, Switzerland; 4 Department of Cytopathology, “Theagenio” Cancer Hospital, Thessaloniki, Greece; 5 Institute of Molecular Biology, Genetics and Biotechnology, Biomedical Research Foundation, Academy of Athens, Athens, Greece; 6 Howard Hughes Medical Institute and Department of Stem Cell and Regenerative Biology, Harvard University and Harvard Medical School, Cambridge, MA, USA

## Abstract

**Background:**

Upregulation of nuclear factor kappa B (NFκB) activity and neuroendocrine differentiation are two mechanisms known to be involved in prostate cancer (PC) progression to castration resistance. We have observed that major components of these pathways, including NFκB, proteasome, neutral endopeptidase (NEP) and endothelin 1 (ET-1), exhibit an inverse and mirror image pattern in androgen-dependent (AD) and -independent (AI) states *in vitro*.

**Methods:**

We have now investigated for evidence of a direct mechanistic connection between these pathways with the use of immunocytochemistry (ICC), western blot analysis, electrophoretic mobility shift assay (EMSA) and proteasome activity assessment.

**Results:**

Neuropeptide (NP) stimulation induced nuclear translocation of NFκB in a dose-dependent manner in AI cells, also evident as reduced total inhibitor κB (IκB) levels and increased DNA binding in EMSA. These effects were preceded by increased 20 S proteasome activity at lower doses and at earlier times and were at least partially reversed under conditions of NP deprivation induced by specific NP receptor inhibitors, as well as NFκB, IκB kinase (IKK) and proteasome inhibitors. AD cells showed no appreciable nuclear translocation upon NP stimulation, with less intense DNA binding signal on EMSA.

**Conclusions:**

Our results support evidence for a direct mechanistic connection between the NPs and NFκB/proteasome signaling pathways, with a distinct NP-induced profile in the more aggressive AI cancer state.

## Introduction

The NFκB transcription factor controls many processes that influence carcinogenesis and cancer progression. Over-expression of NFκB and its transcribed genes are involved in tumor growth, angiogenesis, metastasis, and appear to be correlated with resistance to chemotherapy, advanced tumor stage, PSA recurrence and pre-surgical PSA levels in PC [1-3]. Indeed, there is recent evidence that IL-6 exposure (an NFκB target gene product) induces neuroendocrine differentiation of PC tumour sub-clones, conveying anti-apoptotic phenotype and resistance to chemotherapy [4,5]. Furthermore, activation of NFκB was proved to be sufficient to maintain androgen-independent growth of prostate and PC by up-regulating androgen receptor action [6].

The ubiquitin-proteasome system (UPS) is an indispensable cellular regulatory machine with proteolytic and non-proteolytic functions affecting many cancer-related processes including cell cycle regulation, oxidation damage control, apoptosis, cell trafficking, DNA repair, transcription and chromatic re-modelling [7-10]. Ubiquitination regulates at least three steps in the NFκB pathway: degradation of IκB, processing of NFκB precursors, and activation of the IKK, the latter both degradation-dependent and -independent [1,7].

Neuropeptides (NPs) are naturally occurring peptides that include endogenous opioids Met- and Leu-enkephalin, substance P, bradykinin, angiotensin 1 and 2, ET-1 and bombesin (BBS)-like peptides. Acting as paracrine hormones, they induce responses in many organ systems [11-13]. In relevance to cancer, NPs act as potent mitogens for many cancer types, including small-cell lung cancer and PC [14,15]. NPs like ET-1 and BBS have been shown to stimulate PC growth and new bone formation *in vitro*[15-20], promote cell migration [21-24] and show potent synergy with other growth factors implicated in PC progression [22,25,26]. Advanced and metastatic PC shows upregulated endothelin type A receptor (ET_A_R) and reduced endothelin type B receptor (ET_B_R) expression, therefore sustaining unattenuated ET_A_R-mediated ET-1 action [17,18], and higher plasma ET-1 levels compared to hormone-naïve cancer [16]. This pattern of receptor expression also predicts recurrence of PC following radical prostatectomy [18]. Similarly, advanced PC overexpresses gastrin-releasing peptide (GRP) family receptors, via which BBS-like peptides exert their action [27-30].

NEP is a cell membrane enzyme that hydrolyzes and inactivates NPs [11-13]. During transition to androgen-independence, NEP was shown to be downregulated or silenced [31], frequently via promoter hypermethylation [32]. Furthermore, it appears that androgen deprivation therapy (ADT) leads to emergence of clones that have downregulated NEP expression, as the latter is transcriptionally activated by androgens [31,33,34]. This decrease in NEP expression leaves unopposed the autocrine and paracrine mitogenic action of NPs to act as an alternative growth pathway for PC cells in a low androgen environment [3,21,22,35].

The NFκB/UPS pathway and the NEP/NPs axis are therefore two systems that have been previously shown to be greatly involved in PC progression [1,3,16,31,36-39], but not investigated for their precise interrelation and dynamics. We have observed that with regard to the steady state pattern between these two pathways *in vitro*, AI cells have increased UPS/NFκB activation and a rich NP milieu due to low NEP activity, while AD cells exhibit an exact mirror image [40]. In this work we have investigated the hypothesis that these pathways are directly linked, and that this link has specific dynamics in PC progression. Elucidation of such biological influences could identify potential benefit from combined clinical targeting of these pathways in castration-resistant, advanced stage PC patients.

## Materials and methods

### Cell culture and reagents

LnCaP, PC-3 and HeLa cell lines were purchased from the European Collection of Cell Cultures (ECACC, Health Protection Agency, Salisbury, UK) and all experiments were performed within six months from purchase. The lines were cultured in RPMI 1640 (Euroclone, UK) supplemented with 10% heat-inactivated FBS (GIBCO, UK), 5% L-glutamine (GIBCO, UK) and 1% penicillin-streptomycin (Euroclone, UK) at 37°C in a humidified 5% CO_2_ atmosphere.

A series of incubations were performed, utilising agonists and antagonists involved in the NEP/NPs and NFκB/UPS pathways. ET-1 peptide and ET_A_R antagonist (BQ-123) were purchased from Phoenix Pharmaceuticals, Inc, Germany. BBS, BB_2_/GRP-preferring receptor antagonist (RC-3095), IKK inhibitor (wedelolactone), NFκB inhibitor (BAY 11–7082), and recombinant human tumor necrosis factor α (rhTNFα) were from Sigma Aldrich, UK. Proteasome inhibitor (Bortezomib, VELCADE®) was purchased from Janssen-Cilag Pharmaceuticals, Greece. Recombinant human NEP enzyme (rhNEP) was a kind offer by Dr David Nanus, Weill Cornell Medical College, New York, USA. Protein quantification was done with the use of the Bradford quantification assay (Bio-Rad Laboratories, Inc.) for the total cell lysates, and the BCA Protein Kit (PIERCE Endogen, UK) for nuclear extracts.

### Immunocytochemistry (ICC)

Cells were spread and cultured on glass slides. When at 80–90% confluency cells were fixed with Merckofix® spray fixative (Merck KGaA, Darmstadt, Germany) and conventional avidin-biotin ICC was performed. The Ventana NexES Automated Slide Stainer and related Ventana reagents were used. The samples were immersed in a citrate buffer solution (pH 7.3) and heated for 15 min at 350 W. They were subsequently incubated with 3% H2O2 for 4 min to quench the endogenous peroxidase activity. A primary antibody against the p65 subunit of NFκB was used in a 1:100 dilution. Diaminobenzidine (DAB) was used as a chromogen for detection of the antigens. Incubation with copper sulfate was performed for enhancement of the colour reaction. The slides were finally counterstained with haematoxylin and coverslipped for examination. A primary antibody against the p65 subunit of NFκB (F-6, mouse monoclonal, Santa Cruz Biotechnology, Inc.) was used.

### Nuclear extracts

Nuclear extracts were prepared as described by Carter et al. [41], with minor modifications as reported previously [42]. 10^6^ cells were washed in cold PBS and collected in 400 μl of ice-cold lysis buffer supplemented with 10 μg/ml of protease inhibitors cocktail and then incubated on ice for 20 min. Nonidet (NP-40) 10% was added to lyse the cells which were vortexed and centrifuged for 20 sec at 4°C at 13,000 rpm. The pellet was resuspended in 100 μl of extraction buffer for 20 min on ice. The nuclear suspension was then centrifuged for 15 minutes at 13,000 rpm and supernatant nuclear extracts stored at −80°C until use.

### Total cell lysates

Total protein cell lysates were prepared using a 0.5% CHAPS buffer, which did not affect proteasomal enzymatic activity. Total lysates were also prepared using a second buffer (containing 10 mM Tris–HCl, 50 mM EDTA, 150 mM NaCl, 1% Triton-X and 10% Glycerol) for western blotting purposes.

### Western blot analysis

30 μg of total protein lysate or nuclear extract of each sample were loaded on 4–12% Bis-Tris polyacrylamide gels and underwent electrophoresis under reducing conditions. Proteins were subsequently transferred on a PVDF blotting membrane. Following blocking with 5% non-fat milk, membranes with incubated with primary antibodies at 4°C overnight. Primary antibodies against the p65 subunit of NFκB (F-6, mouse monoclonal and C-20, goat polyclonal Santa Cruz Biotechnology, Inc.), IκBα (C-15, rabbit polyclonal, Santa Cruz Biotecnhology, Inc.) and actin (clone AC-40, mouse monoclonal, Sigma Aldrich, UK) were used. Secondary antibodies incubations were at a dilution of 1:2500 for 2 hours at room temperature. Chemiluminescence detection (ECL detection reagent, Amersham Biosciences) with autoradiography was used. Densitometric analysis of the bands in blots was performed with the public domain software for image analysis ‘ImageJ’ (National Institute of Health, USA) [43]. All bands were measured in optical density (OD) units and the mean densitometry values of differences in protein expression of three independent experiments are presented as NFκB/actin or IκBα/actin ratios respectively compared to baseline. (Western blot bands are representative but not identical to the densitometry values). Results are also presented in graphical form as a percentage of the mean baseline NFκB/actin or IκBα/actin ratios respectively.

### Electrophoretic mobility shift assay

As NFκB consensus oligonuclotide we used the PRDII element [44] of human IFN-beta enhancer (5′-TGGCCAACATGGTGAAACCCCGTTTCTACT-3′) (IMBB, Microchemistry Laboratory), labelled with [γ-32P] ATP using T4 polynucleotide kinase. The probe was purified through G50 columns (Amersham Biosciences) and then EMSA was performed. Briefly, 3 µg of nuclear extracts were incubated at room temperature for 20 min with 100 ng of labelled double-stranded oligonucleotide in the presence of 20 ng of PolydI-dC (PIERCE Endogen, UK) and 20 µg of BSA. Nuclear extracts from HeLa cells (ATCC, UK) 6 hours post-infection with the Sendai paramyxovirus (Cantell strain) and following incubation with TNFα for 1 hour were used as positive controls. For the supershift assay, nuclear extracts were incubated with 1 µg of anti-p65 rabbit polyclonal antibody (Santa Cruz Biotechnology, Inc.) for 30 min at 4°C, before the addition of the probe. In every case, the protein-DNA complexes were separated on a 7% non-denaturing polyacrylamide gel and bands were visualized using a Taeffun Phospor-Imager/Scanner with the ImageQuant™ TL analysis software (Amersham Biosciences).

### 20S proteasome activity assay

Chymotryptic activity of the 20S proteasome in total cell lysates was measured with a commercially available assay based on the detection of fluorophore 7-Amino-4-methylcoumarin (AMC) (Chemicon International, USA), and was confirmed with the use of an AMC-based in-house protocol assay. All experiments were performed in quadruplicate and measured using a Wallac Victor™ multilabel counter with 380 nm excitation and 490 nm emission wavelengths. Baseline enzyme activity was expressed as RFU/μg of total protein. Values were compared against a fluorogenic substrate (LLVY-AMC) standard curve and a 20 S proteasome control activity curve. Changes in proteasomal activity were expressed as percentage increase or decrease from baseline activity of each line.

### Statistical analysis

All data are expressed as the mean ± SEM of 3 or more experiments, as indicated. The Graph Pad Instat Statistical package for Windows was used. The one-way analysis of variance (ANOVA) with the Bonferroni post-test was used for the comparison of data, and the statistical significance limit was set at p < 0.05.

## Results

### NPs induce IκB-dependent activation of NFκB

**Figure 1:**
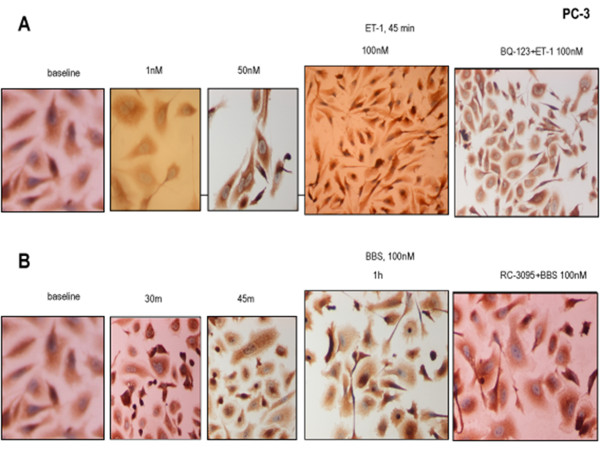
**NP effect on NFκB subcellular localisation.** NFκB expression by ICC. (**A**) PC-3 cells, from left to right: baseline; ET-1 1nM, 50 nM and 100 nM, all incubations x 45 min; BQ-123 (1 μM x 30 min) and ET-1 (100nM x 45 min). (**B**) PC-3 cells, from left to right: baseline; BBS 100 nM, incubations for 30 min, 45 min and 1 h; RC-3095 (10nM x 1 h) and BBS (100nM x 1 h).

We have previously shown that both ET-1 and BBS stimulation at a dose of 100 nM for 60 min resulted in increased nuclear NFκB amount in PC-3 but not in LnCaP cells [45]. In this study, incubation of PC-3 cells with different concentrations of ET-1 showed no appreciable effect on NFκB localization at low concentrations (1nM or 50nM). However, higher ET-1 concentration (100nM) resulted in nuclear translocation of NFκB in the great majority of cells. This effect was time-dependent, evident at the 30-minute incubation and gradually intensifying at the 45-and 60-minute incubation (Figure 1A).

Nuclear translocation in PC-3 cells was also achieved with the use of BBS. As BBS was already used by others [23] to successfully achieve nuclear translocation in PC-3 cells at a specific incubation protocol (100nM for 30 min), we designed a time-series (30, 45, 60, 120 minutes) for the same BBS concentration (100nM). Similar to endothelin, BBS induced a time-dependent nuclear translocation of NFκB, which started at 30 minutes, intensified at 45 minutes and peaked at 1 hour (Figure 1B).

**Figure 2:**
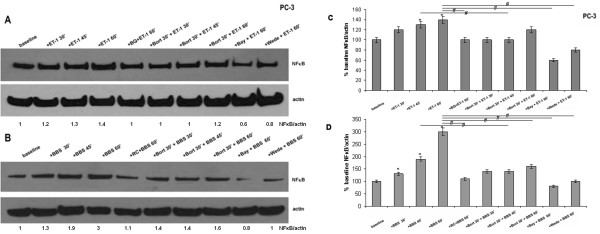
**NP effect on nuclear NFκB amount.** Nuclear extracts blots. (**A**) PC-3 cells, from left to right: baseline; ET-1 incubations (100 nM) x 30 min, 45 min and 60 min; BQ-123 (1 μM x 30 min) and ET-1 (100 nM x 60 min); pre-incubation with bortezomib (1 μM x 30 min) followed by co-stimulation with ET-1 (100 nM) x 30 min, 45 min and 60 min; pre-incubations with BAY 11–7082 (20 μM x 16 h) and wedelolactone (50 μM x 90 min) followed by ET-1 incubations (100 nM x 1 h). (**B**) PC-3 cells, from left to right: baseline; BBS incubations (100 nM) x 30 min, 45 min and 60 min; RC-3095 (10 nM x 1 h) and BBS (100 nM x 1 h); pre-incubation with bortezomib (1 μM x 30 min) followed by co-stimulation with BBS (100 nM) x 30 min, 45 min and 60 min; pre-incubations with BAY 11–7082 (20 μM x 16 h) and wedelolactone (50 μM x 90 min) followed by BBS incubations (100 nM x 1 h). (**C**, **D**) Summary histograms of mean relative band densities for (**A**) and (**B**) normalized to actin expressed as a percentage of baseline (*p < 0.05 versus baseline, ^#^p < 0.05 between conditions as indicated by horizontal lines).

Western blot analysis of nuclear extracts concurred with the ICC results (Figure 1A, B), showing higher nuclear NFκB amount following NP stimulation in PC-3 cells (Figure 2A, B).

**Figure 3:**
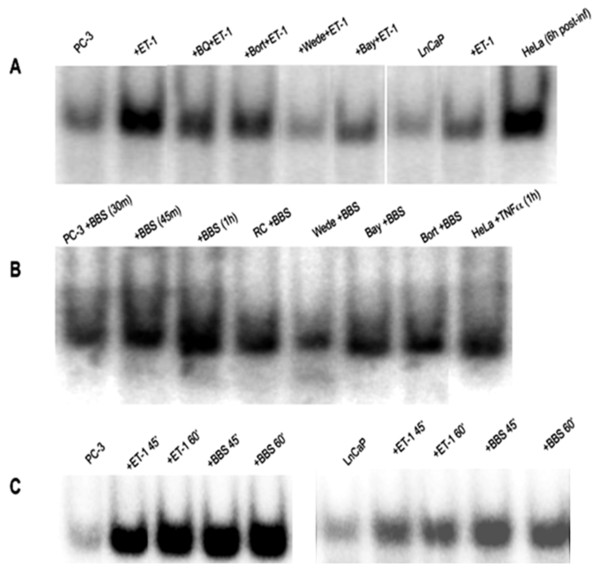
**NP effect on NFκB-DNA binding.** EMSA. (**A**) From left to right: PC-3 cells, baseline; PC-3 cells, ET-1 incubation (100 nM x 45 min); PC-3 cells, pre-incubations with BQ-123 (1 μM x 30 min), bortezomib (1 μM x 60 min), wedelolactone (50 μM x 90 min) and BAY 11–7082 (20 μM x 16 h) followed by ET-1 incubation (100 nM x 45 min); LnCaP cells, baseline; LnCaP cells, ET-1 incubation (100 nM x 45 min); HeLa cells, infection with Sentai paramyxovirus (Cantell strain) for 6 h. (**B**) From left to right: PC-3 cells, BBS incubations (100 nM) x 30 min, 45 min and 60 min; pre-incubation with RC-3095 (10 nM x 1 h), wedelolactone (50 μM x 90 min), BAY 11–7082 (20 μM x 16 h) and bortezomib (1 μM x 60 min) followed by BBS incubations (100 nM x 1 h); HeLa cells, rhTNF-α incubation (10 μM x 1 h). (**C**) From left to right: PC-3 cells, baseline; ET-1 incubations (100 nM) x 45 min and 60 min; BBS incubations (100 nM) x 45 min and 60 min. LnCaP cells, baseline; ET-1 incubations (100 nM) x 45 min and 60 min; BBS incubations (100 nM) x 45 min and 60 min.

In order to demonstrate whether the observed nuclear translocation of NFκB corresponded to actual transcriptional activation effect, we performed EMSA analysis. NP stimulation resulted in increased intensity of NFκB binding signal in a time-dependent manner (Figure 3B, C) similar to the one observed at ICC (Figure 1B). Peak signal intensity was comparable to signal intensity of PC-3 cells treated with TNFα, a well-known activator of NFκB, as well as the signal intensity exhibited by HeLa cells following TNFα incubation or viral infection as above (Figure 3A, B).

**Figure 4:**
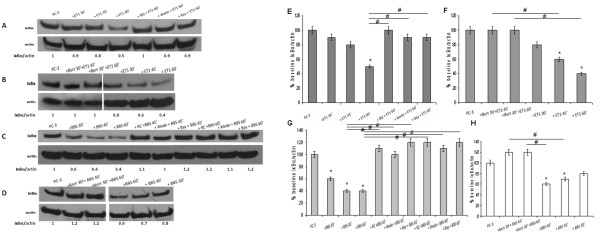
**NP effect on cellular IκBα amount.** Total cell lysate blots. (**A**) PC-3 cells, from left to right: baseline; ET-1 incubations (100 nM) x 30 min, 45 min and 60 min; pre-incubations with BQ-123 (1 μM x 30 min), wedelolactone (50 μM x 90 min) and BAY 11–7082 (20 μM x 16 h) followed by ET-1 incubation (100 nM x 45 min). (**B**) PC-3 cells, from left to right: baseline; pre-incubation with bortezomib (1 μM x 30 min) followed by co-stimulation with ET-1 (100 nM) x 45 min and 60 min; ET-1 incubations (100 nM) x 30 min, 45 min and 60 min. (Different parts of the same gel). (**C**) PC-3 cells, from left to right: baseline; BBS incubations (100 nM) x 30 min, 45 min and 60 min; pre-incubations with RC-3095 (10 nM x 1 h), wedelolactone (50 μM x 90 min) and BAY 11–7082 (20 μM x 16 h) followed by BBS incubation (100 nM x 45 min); pre-incubations with RC-3095 (10 nM x 1 h), wedelolactone (50 μM x 90 min) and BAY 11–7082 (20 μM x 16 h) followed by BBS incubation (100 nM x 45 min). (**D**) PC-3 cells, from left to right: baseline; pre-incubation with bortezomib (1 μM x 30 min) followed by co-stimulation with BBS (100 nM) x 45 min and 60 min; BBS incubations (100 nM) x 30 min, 45 min and 60 min. (Different parts of the same gel). (**E**-**H**) Summary histograms of mean relative band densities for (**A**-**D**) normalized to actin expressed as a percentage of baseline (*p < 0.05 versus baseline, ^#^p < 0.05 between conditions as indicated by horizontal lines).

NP incubation of PC-3 cells also resulted in a time-dependent change in total IκBα status. IκBα levels were already decreased at the 30-minute incubation, and continued dropping at longer incubations (up to 60 minutes) (Figure 4A, B, C, D).

**Figure 5:**
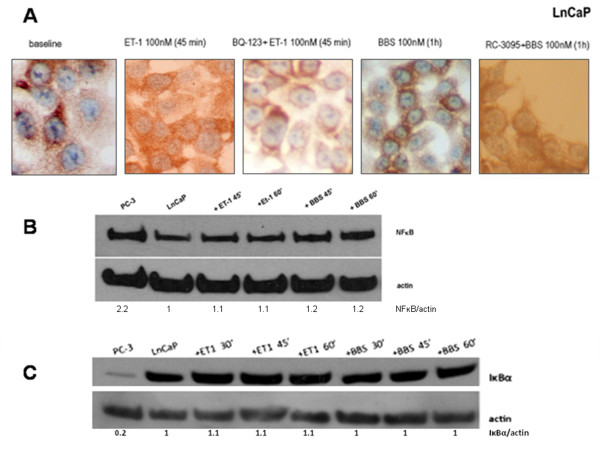
**NP effect on NFκB subcellular localisation and cellular NFκB, IκBα amount.** NFκB expression by ICC and nuclear extract blots. IκBα expression by total cell lysate blots. (**A**) From left to right: LnCaP cells, baseline; LnCaP cells, ET-1 (100 nM x 45 min); LnCaP cells, BQ-123 (1 μM x 30 min) and ET-1 (100 nM x 45 min); LnCaP cells, BBS (100 nM x 1 h); LnCaP cells, RC-3095 (10 nM x 1 h) and BBS (100 nM x 45 min). (**B**) From left to right: PC-3 cells, baseline; LnCaP cells, baseline; LnCaP cells, ET-1 incubations (100 nM) x 45 min and 60 min; BBS incubations (100 nM) x 45 min and 60 min. (**C**) From left to right: PC-3 cells, baseline; LnCaP cells, baseline; ET-1 incubations (100 nM) x 30 min, 45 min and 60 min; BBS incubations (100 nM) x 30 min, 45 min and 60 min.

NP stimulation using the same time and concentration protocols had a relatively small effect on NFκB localization in LnCaP cells, which remained cytoplasmic (Figure 5A), and resulted in minimal increase in nuclear NFκB amount (Figure 5B). Being a more sensitive technique, EMSA analysis showed enhanced NFκB binding signal in LnCaP cells after NP incubations, but to a lesser degree compared to PC-3 cells (Figure 3A, C).

NP incubation resulted in no discernible decrease in total IκBα amount in LnCaP cells at any incubation time (Figure 5C) while there was a profound decrease in PC-3 cells (Figure 4A, B, C, D). It is also obvious that the NFκB/IκBα ratio is much higher than 1.0 in PC-3 while is exactly the opposite in LnCaP where the ratio is lower than 1.0.

### NP-induced NFκB activation is blocked by UPS/NFκB inhibitors

**Figure 6:**
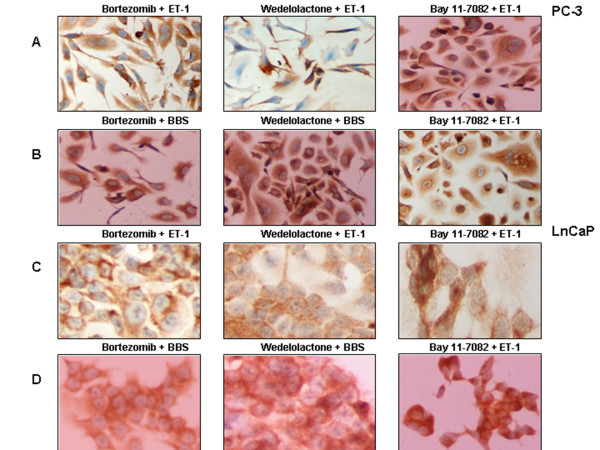
**UPS/NFκB pathway inhibitors prevent NP-induced nuclear translocation of NFκB. NFκB expression by ICC.** (**A**) PC-3 cells, from left to right: incubation with ET-1 (100 nM x 45 min) after pre-incubations with bortezomib (1 μM x 60 min), wedelolactone (50 μM x 90 min), and BAY 11–7082 (20 μM x 16 h). (**B**) PC-3 cells, from left to right: incubation with BBS (100 nM x 1 h) after pre-incubations with bortezomib (1 μM x 60 min), wedelolactone (50 μM x 90 min), and BAY 11–7082 (20 μM x 16 h). (**C**) LnCaP cells, from left to right: incubation with ET-1 (100 nM x 45 min) after pre-incubations with bortezomib (1 μM x 60 min), wedelolactone (50 μM x 90 min), and BAY 11–7082 (20 μM x 16 h). (**D**) LnCaP cells, from left to right: incubation with BBS (100 nM x 1 h) after pre-incubations with bortezomib (1 μM x 60 min), wedelolactone (50 μM x 90 min), and BAY 11–7082 (20 μM x 16 h).

The UPS/NFκB inhibitors also blocked BBS– and ET-1–induced nuclear translocation of NFκB in PC-3 cells. Specifically, pre-incubation with proteasomal and IKK inhibitors (bortezomib and wedelolactone respectively) effectively prevented translocation, as did pre-incubation with the NFκB inhibitor (BAY 11–7082). The latter also had a visible effect on cell morphology (scattered, round, apoptotic looking) as observed in previous incubations (Figure 6A, B).

UPS/NFκB inhibitors also blocked the expected NP-induced increase in nuclear NFκB amount, concurring with the above (Figure 2A, B). Similarly, EMSA analysis showed prevention or attenuation of NP-induced NFκB binding with pre-incubations with the UPS/NFκB inhibitors for both NPs (Figure 3A, B). Finally, pre-incubations with proteasome (Figure 4B, D), IKK and NFκB (Figure 4A, C) inhibitors also blocked the reduction in IκBα amount documented with NP incubations alone.

As expected, pre-incubation with UPS/NFκB inhibitors followed by NP stimulation had no effect on LnCaP cells with regard to NFκB activation (Figure 6C, D).

### NP-induced NFκB activation is blocked by NP receptor antagonists and NEP

**Figure 7:**
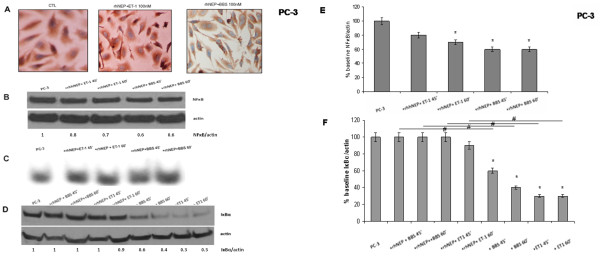
**rhNEP prevents NP-induced NFκB activation.** (**A**) NFκB expression by ICC. PC-3 cells, from left to right: baseline, rhNEP (50 μg/ml x 16 h) and ET-1 (100 nM x 45 min); rhNEP (50 μg/ml x 16 h) and BBS (100 nM x 1 h). (**B**) Nuclear NFκB blot. PC-3 cells, from left to right: baseline; rhNEP (50 μg/ml x 16 h) followed by ET-1 incubations (100 nM) x 45 min and 60 min; rhNEP (50 μg/ml x 16 h) followed by BBS incubations (100 nM) x 45 min and 60 min. (**C**) EMSA. PC-3 cells, from left to right: baseline; rhNEP (50 μg/ml x 16 h) followed by ET-1 incubations (100 nM) x 45 min and 60 min; rhNEP (50 μg/ml x 16 h) followed by BBS incubations (100 nM) x 45 min and 60 min. (**D**) Total cellular IκBα blot. PC-3 cells, from left to right: baseline; rhNEP (50 μg/ml x 16 h) followed by BBS incubations (100 nM) x 45 min and 60 min; rhNEP (50 μg/ml x 16 h) followed by ET-1 incubations (100 nM) x 45 min and 60 min; BBS incubations (100 nM) x 45 min and 60 min; ET-1 incubations (100 nM) x 45 min and 60 min. (**E**, **F**) Summary histograms of mean relative band densities for (**B**) and (**D**) normalized to actin expressed as a percentage of baseline (*p < 0.05 versus baseline, ^#^p < 0.05 between conditions as indicated by horizontal lines).

The above noted nuclear translocation that ET-1 induced in PC-3 cells was completely prevented with pre-incubation with a selective antagonist of ET_A_R (Figure 1A). Translocation was partially prevented by pre-incubation with the BB_2_ receptor inhibitor (Figure 1B). When PC-3 cells were pre-incubated with rhNEP, subsequent incubation with either NP failed to result in visible nuclear translocation (Figure 7A). Pre-incubations with NP receptor antagonists and rhNEP also blocked the increase in nuclear NFκB amount induced by NP incubations alone. (Figure 2A, B, 7B).

In the same context, the NP-induced increase in NFκB binding described above was partially prevented by pre-incubation with the ET_A_R antagonist or BB_2_ receptor inhibitor (Figure 3A, B). Similarly, pre-incubation with rhNEP resulted in significant attenuation of the previously noted increase in NFκB binding signal (Figure 7C).

Finally, pre-incubation with ET_A_ and BB_2_ receptor inhibitors and rhNEP all prevented the NP-induced reduction in total cellular IκBα amount (Figure 4A, C, 7D).

### Upregulation of 20 S proteasome activity is an early effect of NP stimulation

We have previously revealed a unimodal pattern of proteasome activity upregulation in a concentration-series of NP-stimulation experiments in PC-3 cells [45].

**Figure 8:**
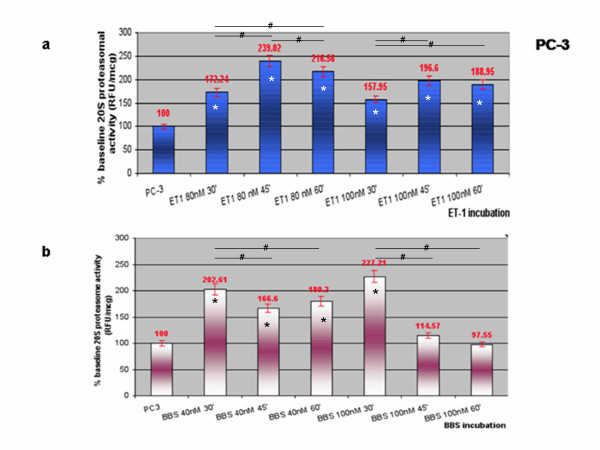
**NP time-dependent effect on 20 S proteasomal activity (RFU/μg) in PC-3 cells.** (**A**) From left to right: PC-3 cells, baseline; time series of ET-1 incubations (80 nM) x 30 min, 45 min and 60 min; time series of ET-1 incubations (100nM) x 30 min, 45 min and 60 min. (**B**) From left to right: PC-3 cells, baseline; time series of BBS incubations (40 nM) x 30 min, 45 min and 60 min; time series of BBS incubations (100nM) x 30 min, 45 min and 60 min. Data represent the mean (± SEM) of three independent experiments performed in triplicate and are expressed as percent of the initial value of proteasome activity (*p < 0.05 versus baseline, #p < 0.05 between conditions as indicated by horizontal lines).

Here, we have further performed time-series of NP-stimulation experiments of the incubations that exhibited peak proteasomal activities (40 nM for BBS and 80 nM for ET-1) as well as of the standard concentration used for the rest of the experiments (100 nM) in PC-3 cells. The ET-1 incubations showed peak activities at 45 minutes (Figure 8A), while the BBS incubations peaked at 30 minutes (Figure 8B).

## Discussion

### NP-inducible NFκB/UPS activation

We have observed that at baseline level there is an inverse expression pattern between the NEP/NPs and NFκB/UPS pathways in AD and AI states *in vitro*[40]. We have also shown that NPs may act as inducers of NFκB activation in PC-3 cells [45]. In the current work we have further investigated whether this mirror phenotype of the two pathways at steady state also translates to a dynamic relationship following dose- and time-dependent stimulation and blocking of the NEP/NPs pathway.

Our evidence shows that increasing concentrations of ET-1 and BBS stimulate translocation of NFκB to the cell nucleus in PC-3 cells (lacking NEP), with resultant activation of NFκB as a transcription factor, evident by increased binding on DNA. Our results concur with these of Levine et al. [23]. They showed that in PC-3 cells BBS induced a time-dependent increase in DNA binding of NFκB peaking at 1 hour which returned to near baseline levels after that. Our results supplemented the above by showing that BBS-induced nuclear translocation is also time-dependent and peaks at the same time as the reported increase in NFκB binding (1 hour), the latter also confirmed in our EMSA results. Furthermore, we have shown that ET-1 has the exact same effect on NFκB activation, evident both at ICC and EMSA analysis.

Our results strongly support that this effect on NFκB activation is due to the NPs themselves and not a chance association. No perceivable nuclear translocation occurred at the same incubations in NEP-expressing LnCaP cells with either ET-1 or BBS. EMSA analysis, being a more sensitive technique did indeed detect a level of NFκB binding activity in these cells, but this was significantly lower compared to PC-3 cells. Similarly, rhNEP incubation successfully prevented nuclear translocation and activation in PC-3 cells.

Further evidence was provided by the fact that this upregulation in NFκB nuclear activity was prevented by the use of the respective receptor inhibitors, and this was shown both via ICC and EMSA. This indicates that this NP-induced effect is receptor-mediated. ET_A_R blockade was predictably able to completely prevent nuclear translocation, as it is established that the main receptor responsible for mediating the mitogenic effects of ET-1 in PC cells is indeed the ET_A_ receptor. BB_2_ receptor blockade was selected for our experiments based on the fact that prostate carcinomas and PC-3 cells in specific are known to abundantly express GRP-R [23,27,30] and that the mitogenic/proliferative effects of BBS in prostate and other types of cancer are predominantly mediated via BB_2_ receptor [28,46]. However, BBS acts on two other receptors, neuromedin B receptor (NMB-R) and BBS receptor subtype 3 (BRS-3), shown to be expressed in 14% and 9% of prostate carcinomas respectively [30]. Levine et al. assumed that BBS-induced NFκB activation is due to activation of GRP-R based on the ability of BB_2_ receptor antagonist to block the BBS-induced increase in intracellular Ca^++^[23]. However, our previously published concentration-series results [45] suggest that the actual NFκB translocation and preceding proteasomal activation is mainly but not completely due to activation of this receptor. Whether blocking of the other two receptors as well would completely prevent nuclear translocation of NFκB remains to be elucidated.

We did not perform a separate analysis of the effect of ET-1 and BBS receptor inhibitors on NFκB at baseline (without NP stimulation). Firstly, a constitutive activation of NFκB has been consistently reported in androgen-independent PC-3 cells, at least partially mediated through epidermal growth factor receptors (EGFR) tyrosine kinases [47], the extracellular signal regulated kinase-1/2 (ERK) [48], NF-κB-inducing kinase (NIK), and IKK activation [38,49]. Therefore, it might seem unlikely to detect a significant effect of NP receptor inhibitors on protein levels and intracellular localization of NFκB at baseline conditions, as NFκB is regulated by multiple signaling pathways which do not necessarily involve upstream NP receptor binding. On the other hand, the use of a specific NP receptor inhibitor in the NEP-expressing LnCaP cells might not offer a significant additional blockage of the mitogenic effects of NPs, including NFκB activation, given that cleavage of NPs by NEP effectively prevents NP receptor binding. In a previous study using BQ-123 at a 10-fold higher dose compared to ours, no effect was observed on baseline secreted levels of IL-6, which is a known NFκB-target gene [50]. In another work, prolonged exposure (72 h) to high doses (25 μM) of another endothelin receptor inhibitor (ABT-627) was needed to produce a discernible effect on NFκB activity [51]. Based on the results of our study examining the stimulated activation of NFκB by ET-1 and BBS, it may be suggested that this is at least partially a receptor-mediated effect as it was reversed by their specific inhibitors.

This effect is associated with increase in proteasomal activity with resultant decrease in IκBα, suggesting that the NP-induced nuclear translocation is IκB-dependent, also prevented by use of NFκB/UPS inhibitors, NP receptor inhibitors and NEP. This suggests that the NP-stimulated NFκB is indeed activated via the canonical pathway.

### NP-induced early proteasomal upregulation model

Our results specifically indicate that NPs are able to upregulate 20 S proteasomal activity at lower concentrations [45] and at shorter incubations than these necessary to achieve NFκB activation. It can therefore be deduced that the increase in proteasomal activity occurs early during NP stimulation and precedes the NFκB nuclear translocation. So it might be that NPs induce proteasomal activity, and when this reaches a critical level it results in NFκB activation via decrease of total IκBα status.

The NP-associated upregulation of proteasomal activity could also explain our finding that ET_A_R antagonist, blocking the action of not only the exogenous but even autocrine- and paracrine-acting ET-1 in PC-3 cells, results in a 50% reduction of baseline proteasomal activity even if it is followed by ET-1 stimulation [45]. The fact that BB_2_ receptor antagonist pre-incubation could not reduce proteasomal activity to lower than baseline [45] could be attributed to the fact that BBS might exert its effect via other receptors as well, as discussed above. It should however be noted that, as the regulation of the proteasome complex activity is a very complicated process, it might not be possible to draw unequivocal conclusions or deduct linear relationships.

LnCaP cells have intrinsic NEP production so paracrine-secreted ET-1 is cleaved. Furthermore, there’s evidence that they have decreased expression of endothelin-converting enzyme 1 (ECE-1), with resultant decrease in production of ET-1 [52]. It is not, therefore, surprising that the effect of exogenous NPs on proteasomal activity is comparably less intense in LnCaP cells [45] or following rhNEP incubation in PC-3 cells. Even at high concentrations, the NP-induced proteasomal activity upregulation [45] does not seem to be strong enough to result in critically low levels of IκBα, thereby not being able to stimulate any visible nuclear NFκB translocation, as we have demonstrated.

The underlying mechanism of NP-induced proteasomal activity upregulation is not known. It could be that NP-mediated increase in IκBα levels results in substrate induction of the proteasome. On the other hand, a direct NP-proteasome interaction or an NFκB-dependent induction of expression of regulatory components of the UPS pathway cannot be excluded and need to be further elucidated. Also, it should be acknowledged as a limitation of this study that our findings were generated only with two different cell lines (LnCaP, PC-3), although it is generally accepted that they do represent preclinical models of AD and AI states, respectively. It is therefore not possible to draw a final conclusion without further studies on other PC cell lines.

## Conclusions

The data presented here suggest that NEP/NP pathway agonists affect the activity of the NFκB/UPS pathway. This is at least partially evidenced by NP-inducible upregulation of proteasome activity which precedes IκB degradation-dependent activation of NFκB in AI PC cells. Recent trends in anticancer therapeutics support the concept of combination therapies for improved efficacy and spectrum of activity. Therefore, translational research into the molecular mechanisms underlying aggressive cancer phenotypes aims towards combined clinical applications of antagonists/inhibitors. As such, combined targeting of the above-mentioned pathways may more effectively prevent survival, invasion and metastasis of PC.

## Abbreviations

AD, Androgen-dependent; ADT, Androgen deprivation therapy; AI, Androgen-independent; AMC, 7-Amino-4-methylcoumarin; BBS, BBS; BCA, Bicinchoninic acid; BSA, Bovine serum albumin; ECACC, European collection of cell cultures; ECE-1, Endothelin converting enzyme 1; ECL, Enhanced chemiluminescence; EMSA, Electrophoretic mobility assay shift; ETAR, Endothelin receptor type A; ETBR, Endothelin receptor type B; FBS, Fetal bovine serum; ET-1, ET-1; GRP, Gastrin-releasing peptide; IFN, Interferon; IκB, Inhibitor kappa B; IKK, IκB kinase; IL-6, Interleukin 6; NEP, Neutral endopeptidase, CD10; NFκB, Nuclear factor kappa B; NP, NP; PBS, Phosphate buffer saline; PC, Prostate cancer; PSA, Prostate-specific antigen; PVDF, Polyvinylidene difluoride; RFU, Relative fluorescence units; rh, Recombinant human; TNFα, Tumor necrosis factor α; UPS, Ubiquitin-proteasome system.

## Competing interests

The authors declare that they have no competing interests.

## Authors’ contributions

CNP and AP designed the study. EH, RMV, CD and EA performed experiments. AP and PJV drafted the manuscript. PJV, IAV and CNP revised the manuscript. All authors read and approved the final manuscript.
